# Evolutionary adaptation of the sensitivity of connexin26 hemichannels to CO_2_

**DOI:** 10.1098/rspb.2016.2723

**Published:** 2017-02-08

**Authors:** Elizabeth de Wolf, Jonathan Cook, Nicholas Dale

**Affiliations:** School of Life Sciences, University of Warwick, Gibbet Hill Rd, Coventry CV4 7AL, UK

**Keywords:** connexin, chemosensitivitiy, breathing, hemichannel

## Abstract

CO_2_ readily combines with H_2_O to form 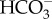 and H^+^. Because an increase of only 100 nM in the concentration of H^+^ (a decrease of 0.1 unit of pH) in blood can prove fatal, the regulated excretion of CO_2_ during breathing is an essential life-preserving process. In rodents and humans, this vital process is mediated in part via the direct sensing of CO_2_ via connexin26 (Cx26). CO_2_ binds to hemichannels of Cx26 causing them to open and allow release of the neurotransmitter ATP. If Cx26 were to be a universal and important CO_2_ sensor across all homeothermic animals, then a simple hypothesis would posit that it should exhibit evolutionary adaptation in animals with different homeostatic set points for the regulation of partial pressure of arterial CO_2_ (PaCO_2_). In humans and rats, PaCO_2_ is regulated around a set point of 40 mmHg. By contrast, birds are able to maintain cerebral blood flow and breathing at much lower levels of PaCO_2_. Fossorial mammals, such as the mole rat, live exclusively underground in burrows that are both hypoxic and hypercapnic and can thrive under very hypercapnic conditions. We have therefore compared the CO_2_ sensitivity of Cx26 from human, chicken, rat and mole rat (*Heterocephalus glaber*). We find that both the affinity and cooperativity of CO_2_ binding to Cx26 have been subjected to evolutionary adaption in a manner consistent with the homeostatic requirements of these four species. This is analogous to the evolutionary adaptation of haemoglobin to the needs of O_2_ transport across the animal kingdom and supports the hypothesis that Cx26 is an important and universal CO_2_ sensor in homeotherms.

## 1. Introduction

In rodents, hypercapnia-evoked release of ATP from the chemosensory areas at the ventral surface of the medulla is physiologically important in the regulation of breathing [1]. Hemichannels of connexin26 (Cx26) are directly sensitive to CO_2_. CO_2_ binds to the hemichannels and causes them to open [2,3] allowing the release of ATP into the extracellular space. In rodents, we have demonstrated that Cx26-mediated ATP release may contribute to the CO_2_-dependent regulation of breathing [4]. In humans, a mutation that removes the CO_2_ sensitivity of Cx26 is accompanied by reduced respiratory drive and periods of central apnoea [5]. It is plausible that Cx26 may also be involved in mediating other physiologically important functions sensitive to CO_2_ such as the control of blood flow.

Theodosius Dobzhansky famously said, ‘nothing in biology makes sense except in the light of evolution’ [6]. If Cx26 is an important and universal mediator of CO_2_ sensitivity in homeotherms, then its CO_2_-binding properties should exhibit evolutionary adaptation in organisms that have differing requirements for the regulation of arterial PCO_2_ (PaCO_2_). It is perhaps not a coincidence that the midpoint of activation of Cx26 by CO_2_ is very close to the resting values of arterial PaCO_2_ in humans −40 mmHg. Birds are well known for their remarkable ability to fly at high altitude. There are numerous anatomical adaptations that ensure efficient uptake of O_2_ from the atmosphere [7,8]. However, these adaptations also mean that birds at altitude need to tolerate very low levels of PaCO_2_ to maintain both their ability to breathe and their cerebral blood flow [9]. Even those birds not known for their ability to migrate at high altitude (such as the chicken) exhibit remarkable tolerance of the effects of low PaCO_2_ on breathing [10,11] and cerebral blood flow [9]. Furthermore, the resting PaCO_2_ of many birds is around 30 mmHg, and in the chicken it has been reported as 33 mmHg [12]. Thus, birds are adapted to a resting PaCO_2_ that is significantly lower than that of humans. By contrast, fossorial mammals such as the naked mole rat live exclusively underground in burrows. These burrows have relatively low gas permeability and thus, through rebreathing of air, become both hypoxic and hypercapnic. Mole rats can thrive when housed in strongly hypoxic and hypercapnic atmospheres, whereas these conditions cause white laboratory rats to lose body weight [13]. CO_2_ equilibrates to a much higher level in subepidermal gas pockets in the mole rat compared with the laboratory rat [14] suggesting that the resting PaCO_2_ of the mole rat may be substantially elevated. Furthermore, mole rats exhibit respiratory responses to changes in inspired CO_2_ over a wider range than the laboratory rat [15].

The physiology of the chicken, human and mole rat therefore provides a sequence to test whether the binding of CO_2_ to Cx26 has been subjected to evolutionary selection pressure. This is, however, a very diverse evolutionary progression. Birds and mammals diverged about 310 Ma, whereas primates diverged from rodents about 100 Ma [16]. Mice and rats last shared a common ancestor with the naked mole rat (*Heterocephalus glaber*) about 73 Ma [17]. Thus, including the rat in our comparison of species gives an additional test between somewhat evolutionarily closer species. Furthermore, the rat has a very similar resting PaCO_2_ to humans [18]. Interestingly, Cx26 in the human, rat and mole rat has more than 90% amino acid identity, whereas the amino acid identity between the chicken and the three mammalian Cx26 proteins is only 74–77% (table 1).

**Table 1.** Amino acid identity (%) of Cx26 from human, rat, mole rat and chicken. NCBI reference sequences: human, NP_003995; rat, NP_001004099.1; mole rat, JAN95966.1 and chicken NP_001257745.1.

	human	rat	mole rat
rat	94		
mole rat	93	92	
chicken	77	76	74

Based on our proposal that Cx26 is a universal sensor of CO_2_ in homeotherms, we propose the simple hypothesis that the sensitivity to CO_2_ of Cx26 should be: highest in birds; at a similar intermediate level in humans and rats; and lowest in mole rats. We have therefore tested this hypothesis by expressing human, chicken (*Gallus gallus*), rat (*Rattus norvegicus*) and mole rat (*Heterocephalus glaber*) Cx26 in HeLa cells. We find that the sensitivity of Cx26 to CO_2_ does indeed follow this predicted progression. This feature of the molecule appears therefore to have been subject to evolutionary selection pressure and supports our hypothesis that CO_2_ sensing via Cx26 plays universal and important physiological roles.

## 2. Material and methods

### (a) Cx26 genes

The coding sequences for Cx26 from each species were taken from the following accession numbers: chicken Cx26, NM_001270816.1; human Cx26, NM_004004.5; mole rat Cx26, XM_004854883.2; rat Cx26, NM_001004099. Cx26 from each species was subcloned into the pCAG-GS mCherry vector prior to mammalian cell transfection. The presence of Cx26 was confirmed by DNA sequencing (GATC Biotech).

### (b) HeLa cell culture

HeLa cells were grown in Dulbecco's modified Eagle's medium (DMEM) supplemented with 10% fetal bovine serum, 50 µg ml^−1^ penicillin/streptomycin and 3 mM CaCl_2_. For dye loading experiments, cells were plated onto coverslips at a density of 5 × 10^4^ cells per well, and transiently transfected with human, chicken, rat or mole rat Cx26 following the GeneJuice Transfection Reagent protocol.

### (c) Artificial cerebrospinal fluid solutions

*Hypocapnic artificial cerebrospinal fluid* (20 *mmHg PCO_2_*). 140 mM NaCl, 10 mM NaHCO_3_, 1.25 mM NaH_2_PO_4_, 3 mM KCl, 10 mM d-glucose, 1 mM MgSO_4_, 2 mM CaCl_2_. This was continuously bubbled with sufficient CO_2_ (approx. 2%, balance O_2_) to give a final pH of approximately 7.4.

*Normocapnic artificial cerebrospinal fluid* (35 *mmHg PCO_2_*). 124 mM NaCl, 26 mM NaHCO_3_, 1.25 mM NaH_2_PO_4_, 3 mM KCl, 10 mM d-glucose, 1 mM MgSO_4_, 2 mM CaCl_2_. This was bubbled with 95%O_2_/5% CO_2_ and had a final pH of approximately 7.4.

*Hypercapnic artificial cerebrospinal fluid* (55 *mmHg PCO_2_*). 100 mM NaCl, 50 mM NaHCO_3_, 1.25 mM NaH_2_PO_4_, 3 mM KCl, 10 mM d-glucose, 1 mM MgSO_4_, 2 mM CaCl_2_. This was bubbled with sufficient CO_2_ (approx. 9%, balance O_2_) to give a final pH of approximately 7.4.

### (d) Dye loading experiments

We used a dye loading protocol that has been developed and extensively described in our prior work [2,3,5,19,20]. HeLa cells expressing Cx26 for 72 h from each of the species tested were initially washed with hypocapnic aCSF. They were then exposed to hypocapnic, normocapnic or hypercapnic aCSF containing 200 µM 5(6)-carboxyfluorescein (CBF) for 10 min. Subsequently, cells were returned to hypocapnic aCSF with 200 µM CBF for 5 min, before being washed in hypocapnic aCSF without CBF for 30 min to remove excess extracellular dye. A replacement coverslip of HeLa cells was used for each condition. For each coverslip, mCherry staining was imaged to verify Cx26 expression.

To ensure valid comparisons were made under as near identical conditions as possible, the measurement of CO_2_ sensitivity of the chicken, and mole rat Cx26 genes was interleaved with the reference gene—human Cx26. Thus, chicken and human comparisons were performed together, and mole rat and human comparisons were performed together. The experiments were replicated independently (independent transfections) five times to give *n* = 5 for chicken, and mole rat and *n* = 10 for human. A separate set of measurements (five independent replications) on rat Cx26 (the variant of Cx26 used in all of our previous papers [2,3,5,20]) was performed to quantify the binding characteristics of CO_2_ to Cx26 in this species.

### (e) Fluorescence imaging and statistical analysis

Following dye loading, HeLa cells were imaged by epifluorescence (Scientifica Slice Scope, Cairn Research OptoLED illumination, 60× water Olympus immersion objective, NA 1.0, Hamamatsu ImagEM EM-CCD camera, Metafluor software). ImageJ [21] was used to measure the extent of dye loading by drawing a region of interest (ROI) around each cell, and subsequently, the mean pixel intensity of the ROI was determined. The mean pixel intensity of a representative background ROI for each image was subtracted from each cell measurement from the same image. At least 50 cells were measured for each condition per experiment, and at least five independent repetitions using the same batch of HeLa cells were completed. The mean pixel intensities were plotted as cumulative probability distributions in figure 2 and these graphs show every data point measured.

To avoid pseudoreplication, statistical analysis was performed (via the scripting language, R) on the medians of the independent replicates with the Kruskal–Wallis ANOVA to compare across all four species and the Mann–Whitney *U*-test for pairwise comparisons between species. Multiple pairwise comparisons were adjusted via the false discovery rate method [22] and only those comparisons that retained significance after this adjustment are reported. In figure 3, the data are presented as the mean, with the error bars being just a positive- or negative-going standard deviation for visual clarity. The mean data points have been fitted with the Hill equation expressed in terms of PCO_2_ and pixel intensity:
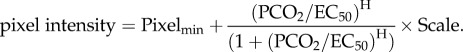


For simplicity, Pixel_min_ (the minimum pixel intensity when hemichannels are fully shut) was set to 1200, and Scale (a scale factor, to determine the maximum pixel intensity) was set to 2900 for all four species. The assumptions underlying this choice are: (i) the background fluorescence would be expected to be the same for all three species, as the experiments were performed under as near identical conditions as possible and (ii) the Cx26 from all four species exhibits the same permeability to CBF and will open fully to a sufficiently high dose of CO_2_. EC_50_ is the PCO_2_ that gives the half maximal effect, and H is the Hill coefficient—a measure of the cooperativity of CO_2_ binding.

## 3. Results

To assess the CO_2_ sensitivity of Cx26 from chicken, human, rat and mole rat, we expressed the relevant Cx26 gene in HeLa cells and used a well-established dye loading assay to examine how uptake of a fluorescent dye, CBF, altered with PCO_2_. Parental HeLa cells have no endogenous connexin hemichannels and do not exhibit CO_2_-sensitive dye loading [2,3,5]. Thus, any dye loading observed in our experiments is due to the functional expression of the exogenous Cx26 gene.

**Figure 1.**
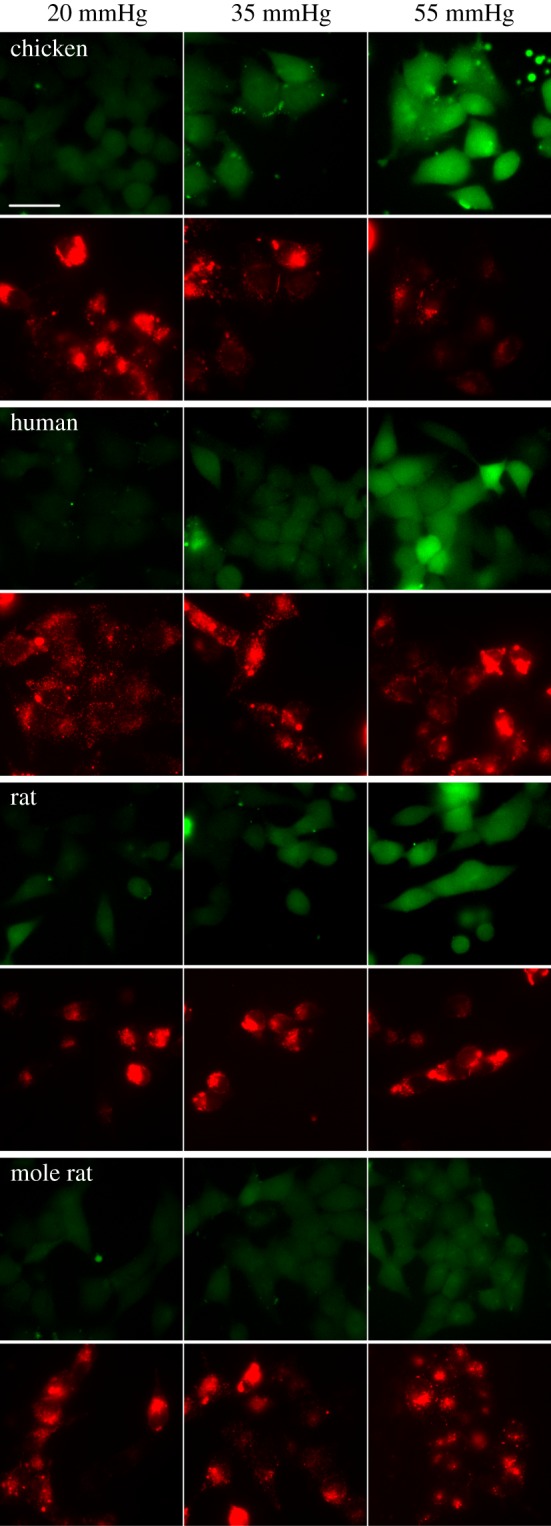
CO_2_-dependent dye loading of HeLa cells expressing Cx26 from chicken, human, rat and mole rat. In each panel, the top images (green) show the CO_2_-dependent loading of 5(6)-CBF at three different levels of PCO_2_. The Cx26 was tagged at the C-terminal with mCherry. The bottom images (red) in each panel show the mCherry fluorescence and hence Cx26 expression corresponding to the cells that were loaded in the images above. Scale bar, 40 µm, applies to all images.

**Figure 2.**
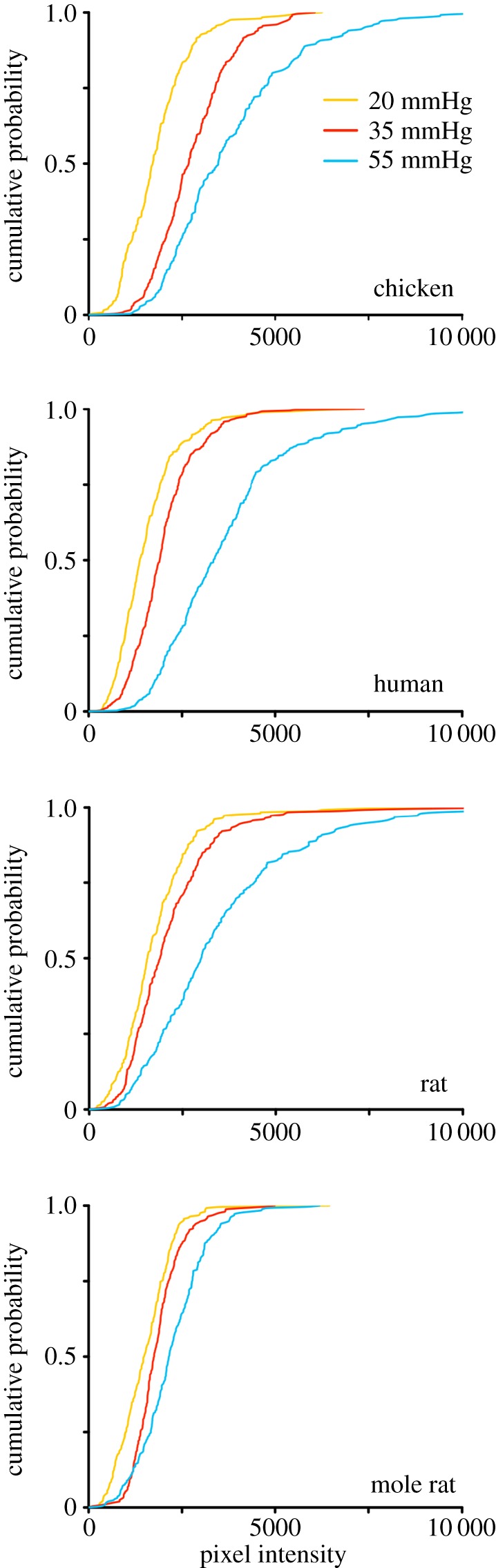
Quantitation of CO_2_ sensitivity of Cx26 from chicken, human, rat and mole rat. Cumulative probability distributions of pixel intensity obtained from images like those in figure 1. For each experiment, 50 cells were measured at each level of PCO_2_. The graphs show all of the data points from five independent replications for chicken, rat and mole rat, and 10 independent replications for human.

We used a PCO_2_ of 20 mmHg as our baseline, and increased PCO_2_ to 35 and 55 mmHg for each species. We found that the baseline dye loading of CBF at 20 mmHg was very similar in the four species (figures 1 and 2, Kruskal–Wallis ANOVA, *p* = 0.8521). At a PCO_2_ of 35 mmHg, the extent of dye loading in the four species differed (figures 1 and 2, Kruskal–Wallis ANOVA, *p* = 0.0062). Pairwise comparisons revealed that chicken Cx26 gave much higher dye loading than human (Mann–Whitney *U*-test, *p* = 0.0013), rat (*p* = 0.0159) and mole rat (*p* = 0.0040). At a PCO_2_ of 55 mmHg, the dye loading also differed between species (figures 1 and 2, Kruskal–Wallis ANOVA, *p* = 0.0057). At this level of PCO_2_, the dye loading into HeLa cells expressing mole rat Cx26 was greatly reduced compared to chicken, human and rat (*p* = 0.0040, *p* = 0.0013, *p* = 0.0159, respectively).

**Figure 3.**
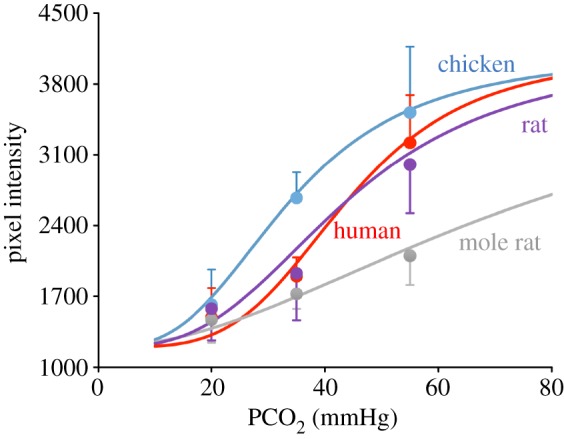
Estimation of the binding characteristics of CO_2_ to Cx26 from chicken, human, rat and mole rat. The data points show the mean pixel intensity at each level of PCO_2_. The error bars are one standard deviation. The curves are the best manual fits of the Hill equation to the data points (see Material and methods and table 2).

To estimate the binding parameters of CO_2_ for Cx26, we assumed that this relationship could be described by the Hill equation (see Material and methods). We plotted the mean dye loading for each species against PCO_2_ (figure 3). Visual inspection showed that both the affinity (EC_50_) and cooperativity (Hill coefficient, H) differed for the four species. Manual fitting gave estimates of the binding parameters (table 2). Interestingly, chicken Cx26 had an EC_50_ of 34 mmHg, which is close to the reported resting PaCO_2_ in chicken (33 mmHg). Mole rat Cx26 had a much higher EC_50_ of 77 mmHg, suggesting that it has been adapted to allow physiological tolerance of high PaCO_2_.

**Table 2.** Estimated binding characteristics of CO_2_ to Cx26 from four different species. Both the EC_50_, a measure of binding affinity, and H, a measure of cooperativity, vary across the species. The differences in affinity reflect the physiological needs of the chicken to tolerate hypocapnia and the mole rat to tolerate hypercapnia. The reduced cooperativity of CO_2_-binding in chicken, rat and mole rat suggests that these species remain sensitive to variations of CO_2_ over a wider range of partial pressures than humans.

	chicken	human	rat	mole rat
EC_50_ (mmHg)	34	44	44	77
H	3	4	3	2

## 4. Discussion

Physiological functions of universal scope across species exhibit evolutionary adaptation to allow organisms to exploit different niches. Often, the properties of the molecules that mediate important physiological roles are subject to evolutionary selection pressure. For example, haemoglobin of the bar-headed goose, which can fly at altitudes up to 9000 m, has a much higher affinity for O_2_ than related low-living species such as the grey lag goose [23]. In this example, there are amino acid substitutions that endow the bar-headed goose haemoglobin with high affinity for O_2_. Andean camelids also have haemoglobin with higher O_2_ affinity and different mutations that affect the binding of allosteric modulators of haemoglobin (2,3-diphosphoglycerate and Cl−) may be important [23]. Overall, the key point is that evolution can fine-tune the properties of universally important molecules in multiple ways to allow them to provide appropriate physiological control for new ecological niches.

In this paper, we have reversed this reasoning, to argue that if we can find evidence of evolutionary adaptation of a molecule involved in homeostatic control then it must be of universal importance. The high metabolic rate of homeothermic animals inevitably results in a high rate of CO_2_ production. For example, adult humans produce approximately 20 moles of CO_2_ per day. As CO_2_ combines with water to produce 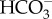 and H^+^, and control of internal body pH is a vital homeostatic function, the regulated excretion of CO_2_ via breathing is of critical importance for life. Therefore, we might expect the key molecules involved in this process to be subjected to evolutionary adaptation across species with different homeostatic requirements. It is of course possible that adaptive changes in the chemosensory regulation of breathing could occur downstream of the chemosensory transducing molecules involved, such as in the neural circuits that control of breathing. These changes could be an additional or completely alternative way of achieving the required alterations in homeostatic control. Nevertheless, adaptation of the properties of the key chemosensitive molecules is a simple and highly attractive hypothesis for evolutionary adaption of the respiratory system.

Our previous work has suggested that in rodents and humans, hemichannels of the gap junction protein Cx26 are direct sensors for CO_2_ that help to regulate breathing [4,5]. We have therefore examined whether the CO_2_-binding properties of Cx26 have been adapted across four species that have a range of resting PaCO_2_ values and tolerances of hypocapnia and hypercapnia. Our results show that the CO_2_-binding properties do indeed vary in a logically consistent manner with the homeostatic requirements of these four species.

The affinity for CO_2_ is higher for chicken Cx26 than human. This corresponds to the observation that in many birds the resting PaCO_2_ is around 30 mmHg. Rat and human Cx26 have a similar affinity for CO_2_ that is quite close to their reported resting values of PaCO_2_. Conversely, the affinity of mole rat Cx26 for CO_2_ is shifted to substantially higher levels of PCO_2_. Estimates of the resting PaCO_2_ of the mole rat appear to be missing from the literature. The only relevant values that we have uncovered are for subcutaneous gas pockets: 86 mmHg under normocapnic conditions and 102 mmHg under hypoxic–hypercapnic conditions found in mole rat burrows [14]. The gaseous composition of subcutaneous gas pockets equilibrates very quickly—presumably with that of capillary blood. Campbell (1931) [24] summarized the stable composition of subcutaneous gas pockets for a number of mammals as having a PCO_2_ in the range 45–52 mmHg. This is somewhat higher than the PaCO_2_ in these animals—approx. 40 mmHg. This would suggest that the PaCO_2_ of mole rat is probably less than the 86 mmHg observed in the subcutaneous gas pocket, but still much higher than that of non-fossorial mammals: i.e. a value around 70–75 mmHg would be plausible. If this is the case, then the EC_50_ of mole rat Cx26 for CO_2_ does likely match its physiological requirement.

Interestingly, the cooperativity of CO_2_ binding appears to differ between the four species. Human Cx26 has the highest cooperativity with a Hill coefficient of 4. Thus, this Cx26 variant responds over a narrow range of PCO_2_. The rat, chicken and mole rat Cx26 have lower cooperativity than human Cx26. They thus respond over a wider range of PCO_2_ and this is particularly marked for mole rat where the Hill coefficient is only 2. This may be particularly significant as breathing in the mole rat responds to changes in inspired PCO_2_ that extend over a much wider range (35–125 mmHg) compared with the laboratory rat and other non-fossorial mammals [15]. The low cooperativity of CO_2_-binding to mole rat Cx26 and resulting sensitivity to a wide range of ambient PCO_2_ would therefore appear to be suitable for the physiological requirements of this species.

**Figure 4.**
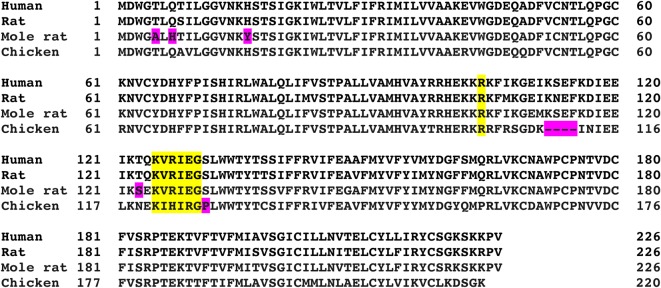
Sequence comparison of human, rat, mole rat and chicken Cx26. The yellow boxes highlight the carbamylation motif. The pink boxes indicate amino acid substitutions in the sequences of mole rat and chicken Cx26 that could contribute to their altered CO_2_ sensitivity compared to human Cx26.

The key modifications of Cx26 in the chicken and mole rat compared to humans that give rise to, respectively, higher and lower CO_2_ sensitivity remain open to speculation. Rat and mole rat Cx26 are very homologous to human Cx26 and to each other (table 1 and figure 4). Thus, very few amino acid changes appear to be required to reduce the affinity of CO_2_ binding in Cx26. Intriguingly, there is a tyrosine in place of p.His16 (figure 4). The mutations p.Asn14Lys and p.Asn14Tyr, which are dominant mis-sense mutations that cause KID syndrome [25,26], abolish the CO_2_ sensitivity of Cx26 in a dominant manner [20]. p.Asn14 is close, in the three-dimensional structure of Cx26 [27], to the CO_2_-binding site (p.Lys125 and p.Arg104) [3]. As p.Tyr16 in mole rat is also close by, it is possible therefore that the presence of this bulky aromatic residue might reduce CO_2_ sensitivity.

Chicken Cx26 is genetically quite different from that of human, rat and mole rat (table 1 and figure 4) but retains the CO_2_-binding motif (albeit highly modified). Interestingly, the cytoplasmic loop is shorter by four amino acids (figure 4) and there is a proline residue (p.Pro127) in the helix just prior to the binding motif. The cytoplasmic loop of Cx26, which has not been resolved in X-ray structures [27,28], is likely to contribute to the environment of p.Lys125 and hence might alter its propensity for carbamylation. Additionally, p.Pro127 might slightly alter the orientation of p.Lys121 in chicken Cx26 towards p.Arg104 and favour carbamylation of this residue at lower levels of PCO_2_. It is also possible that the highly modified CO_2_-binding motif may also alter the sensitivity of CO_2_-binding. Only deeper structural understanding of Cx26, tested by targeted mutagenesis of key residues, will enable us to distinguish these possibilities.

Cx26 is a multifunctional molecule—it not only forms hemichannels, but also gap junctions, which are important in many physiological contexts outside the control of breathing or blood flow. Thus, any changes in amino acid sequence that affected gap junction or hemichannel permeability, gap junction stability, the ability of individual hemichannels (connexons) to dock together to form gap junctions or the gating of hemichannels by extracellular Ca^2+^ would likely be highly maladaptive. However, the CO_2_-binding site in Cx26 [3] is well away from the lining of the pore and putative Ca^2+^-binding sites [28], and is located at the intracellular surface of the channel far away from the connexon docking site [27], which is on the extracellular surface. Thus, these constraints on possible amino acid alterations within Cx26 may not be too limiting for adaptation of CO_2_-binding properties. Nevertheless, rigorous structural understanding of what determines the affinity of the binding site for CO_2_ is conspicuously absent.

So far, we have framed our discussion in the context of Cx26 as a universal CO_2_ sensor in homeotherms. Fish and gill-breathing amphibians mainly regulate ventilation in response to changes in PO_2_ [29]. There is evidence for sensitivity to PCO_2_ in fish, but this is over a much lower concentration range than in mammals and is a relatively minor contribution to the regulation of breathing compared to PO_2_ [30–32]. The prime importance of CO_2_ in respiratory control arises in air-breathing vertebrates—lung fish [33,34], post-metamorphic amphibians, reptiles and higher organisms [31,32]. Interestingly, lung fish appear to have chemosensors that are independently sensitive to pH and PCO_2_ and are thus similar to mammals in this respect [34]. No Cx26 orthologues have been reported in fish, but Cx26 orthologues are present in amphibians [35].

Connexins are found only in chordates, yet many invertebrates, e.g. *C. elegans* [36,37] and insects [38], can detect CO_2_ for aversive purposes or for location of host species or food. In these latter examples, it appears that CO_2_-sensitive GPCRs are involved. The role of connexins in forming gap junctions is taken by the unrelated innexin gene family in invertebrates. The innexins are homologous to the pannexin genes of vertebrates [39], but we are not aware of any evidence to suggest that either innexins or pannexins are CO_2_-sensitive.

In summary, we report that the CO_2_-binding properties of Cx26, a connexin that coincidentally arose around the time of the evolution of air-breathing animals, are adapted to suit the physiological homeostatic requirements of an evolutionarily wide range of homeotherms. This suggests that it may indeed be a universal CO_2_ sensor in this group of animals.

## Supplementary Material

Raw data for mole rat

## Supplementary Material

Raw data for human

## Supplementary Material

Raw data for rat

## Supplementary Material

Raw data for chicken

## Supplementary Material

Median data

## Supplementary Material

Multiple comparisons

## Data accessibility

The data supporting this article have been uploaded as part of the electronic supplementary material.
